# Emotional and behavioral attitudes of Tunisian youth towards childhood leukemia: health education and primary prevention in perspective

**DOI:** 10.1186/s12889-022-14596-6

**Published:** 2022-11-17

**Authors:** Foued Maaoui, Imen Moumni, France Arboix-Calas, Ines Safra, Samia Menif

**Affiliations:** 1 Laboratory of molecular and cellular hematology, Pasteur Institute of Tunis, Tunis, Tunisia; 2 ISEFC, Virtual University of Tunis, Tunis, Tunisia; 3 Faculty of Education, University of Montpellier, Montpellier, France

**Keywords:** Environmental health literacy, Leukemia, Secondary school students, Attitudes, Environmental risk factors, Tunisia

## Abstract

**Background:**

Given the increasing blood cancer incidence in Tunisia and recent discoveries proving the involvement of environmental factors, this study examined the environmental health literacy (EHL) of Tunisian secondary school students concerning not only this disease, but also their emotional and behavioral attitudes towards leukemia risks.

**Methods:**

A cross-sectional survey was conducted among Tunisian youths (*N* = 372, 16–20 years; 68% females, 32% males). Data collection took place in four representative public secondary schools in the North, Center, and South of Tunisia. Students completed a paper and pencil questionnaire and described their EHL level of blood cancer, as well as their attitudes and interests in this disease. The statistical software (SPSS, v.25.0) was used to analyze the data collected.

**Results:**

The results indicated low EHL levels of leukemia. Most youths failed to identify all the leukemogenic (except tobacco and pollution) and non- leukemogenic risk factors. Pesticide use and exposure to low frequency electromagnetic radiation were not considered risk factors. Proximity to heavy-traffic roads and benzene exposure were not perceived by youth as risk factors. Despite these low levels, most participants were interested in having more information about leukemia and cancers in general.

**Conclusion:**

This investigation shows a lack of knowledge about leukemia. Low EHL levels will incite educational actors and curriculum designers to optimize content and innovate ICT adapted to this environmental health challenge.

## Introduction

Leukemia is one of the major public health problems worldwide and in developing countries with 10,794 new cases in 2020 and a mortality of 3.6 in North Africa, 617 new cases in Tunisia with an incidence of 4.6, a mortality of 3 and a prevalence of 14.2 per 100,000 inhabitants in 2020. In the case of Tunisia, the international agency for research on cancer predicts an increase in deaths from 442 in 2020 to 817 in 2040 [1].

As a result, lifestyle choices such as smoking, physical inactivity, consumption of energy foods and certain practices (the use of domestic and agricultural insecticides, proximity to heavy-traffic roads, benzene exposure, and other sources of low-frequency electromagnetic fields) have increased the number of leukemia cases [2–4].

Indeed, there is an extensive literature demonstrating the impact of environmental agents on determining the type of leukemia.

In the case of childhood leukemia, the evidence implicating environmental causes comes from a variety of studies including meta- and pooled analyses. Exposures to agents like pesticides, tobacco smoke, solvents, and traffic-related pollution have been consistently associated with an increased risk of developing childhood leukemia. On the other hand, multivitamin and folic acid supplementation during preconception or pregnancy has been linked to a reduced risk of childhood leukemia [5, 6], as it has breastfeeding and early exposure to infection characterized by attendance at daycare and preschool settings.

Furthermore, risk factors for childhood leukemia are also associated with the risk of developing other cancers, neurobehavioral deficits, and respiratory disorders [7].

Despite the fact that many studies have identified modifiable risk factors (increased or reduced risk) for childhood leukemia, we are aware of no current Tunisian prevention program that specifically deals with leukemia. However, several researchers have shown that health communication and education have a positive effect on knowledge and attitudes towards health problems [8].

Public education is an important component of cancer control programs and has been shown to be effective in bringing about lifestyle changes [9].

The World Health Organization (WHO) recognizes the importance of education in raising cancer awareness, changing specific risk behaviors (such as smoking cessation), learning self-examination techniques (such as breast self-examination), and promoting early cancer detection in the community [9].

The literature reports that educational programs resulted in the change in the individual’s knowledge, attitudes and beliefs regarding cancer [10].

Different papers described the effectiveness of health education in creating an intention for change, awareness, perceived susceptibility and the severity or seriousness of this disease [11].

They proved that health promotion and education resulted in the increase of knowledge, change in the attitude and intention for cancer screening, perceived self-efficacy, reduction of social obstacles for adopting the healthy behavior, timely diagnosis of cancer, change of behavior and reduction of cancer risk factors.

A randomized trial was designed to evaluate a short educational intervention for cancer prevention. The promotion of a balanced diet in French school children demonstrates the effectiveness of education in changing behaviors [12].

Barros et al. (2014) proved the importance of cancer education using a quantitative study. The study showed the success of a training program, entitled “Cancer, Educate to Prevent”, that allowed teachers to independently develop and implement prevention campaigns focused on students and school-related communities.

The program included different educational modules, ranging from cancer biology to prevention campaign design. The results showed a significant improvement in the perception and knowledge about cancer among teachers and students [13].

A randomized controlled trial of the impact of a web-based intervention supplemented with text messages to improve cancer prevention behaviors among adolescents showed some improvements in students’ weight control after 9 months of intervention [14].

The results of these studies demonstrate the importance of education in reducing exposure to cancer risk factors. Health education is a singular form of health communication as it is directed to youths.

The objectives surpass the mere fact of informing or sensitizing people to cancers. It is mainly about constructing a reasonable environmental risk perception by developing capacities to access reliable information, criticize this information and make healthcare decisions.

Furthermore, the specific characteristics associated with each disease induce an emotionally charged individual, whose intensity modulates one’s perception of the danger as well as actions to avoid or reduce it. This applies to cancers. Being aware of one’s emotions and how to regulate them will determine each individual’s behaviors towards risk factors and situations.

In developing countries, special focus is put on resisting social norms and cultural pressures. Cancer education is therefore about acquiring knowledge and developing life skills.

### What is the representation of leukemia by Tunisian students?

Various psychological theories have been applied for preventive purposes. Among these theories, the health belief model, the theory of planned behavior and the trans-theoretical model of behavior change have been used for cancer prevention [15].

In this study, we have used the Common-Sense Model (CSM) of self-regulation (Leventhal et al., 1997) to evaluate knowledge, leukemia representations, preventive intentions and skills for the management of environmental risk factors.

The latter, developed by Howard Leventhal, Daniel Meyer and David R. Nerenz (1980), is a combination of two major behavior theories in health psychology: behavioral theories and social-cognitive and emotional theories [16].

According to CSM, the disease representation stored in memory can be defined as a synthesis of the individual’s own knowledge about the disease, information from recognized external sources, and personal experiences of the disease and its symptoms.

In our theoretical framework sections (1.1, 1.2 and 1.3), we explain how the CSM enables the integration of concepts about emotions, risk perception and health literacy.

The use of this conceptual model, allowed us to analyze in depth the factors affecting preventive behaviors and intentions of our students.

#### Three postulates founding the common-sense model (CSM)

1.The individual is thought of as an active agent solving problems related to his/her health. In our case, students will try to reduce the gap between their perceptions of leukemia and the scientific knowledge about the disease by seeking to understand how to avoid this disease.
In the healthcare field, the lay representations of individuals are often opposed to the scientific representations conveyed by the medical profession. They are personal representations that are constructed on the basis of the individual’s own experience of the illness, the discourse of trusted third parties (peers, family, community) and the scientific discourse.
The constructed representation of the disease leads to its adaptation and then the individual will proceed to the subsequent evaluations which confer the dynamic character of the model.

#### Five dimensions of illness representation

The CSM evokes both behavioral and cognitive parallel responses to the individual’s constructed illness representation in which the five dimensions can be assessed [17, 18].

1. Identity: the label or name given to the disease and its associated symptoms. It determines the perceived severity of the disease.
2. Cause: the set of personal ideas about the causes of the disease even if they are not scientifically correct.
3. Chronology: predictive beliefs regarding the expected duration of the disease.
4. Consequences: personal representations on the consequences of the disease and their impact on human life.
5. Controllability: beliefs about the possibility of avoiding or curing the disease.

In this study, four CSM dimensions were mobilized in order to not only assess Tunisian students’ knowledge of environmental factors associated with leukemia (causes), but also identify affective determinants of attitudes and behaviors (Identity, Consequences and Controllability). Table 1.

**Table 1:** Adequacy between leukemia representation, CSM dimensions and questionnaire items

Leukemia representation	CSM dimensions	Objects	Items
Knowledge (Fig. 1)	Cause	Environmental factors	A1, B1, C1, D1, E1, G1, H1, K1
Affective determinants of attitudes and behaviors (Table 2)	Identity	Fear	I
	Consequences	Depression	F
		Anxiety	G
		Sadness	H
		Negative thoughts	J
	Controllability	Communication	M, N, O
		Lifestyle choices	P, Q
		Social engagement	R, S

### Emotions and risk perception as antecedents to preventive behaviors

Rimal and al. (1999; 2001) revealed that “when people recognize that they are vulnerable to the risk, they become motivated to adopt preventive behaviors” [19, 20].

In this perspective, several health behavior models, such as the “Health Belief Model” [21, 22], the “Protective Motivation Theory” and the “Precautionary Coping Process Model” have been proposed [23–28]. According to Van der Pligt (1996) and Oh (2021), all these models share the same view that “the higher perceived health risk motivates people to adopt preventive behaviors” [29, 30].

Turner (2012) and Tsoy (2021) confirm that “emotions can not only affect behavioral outcomes via risk perception but can also directly motivate preventive behaviors” [31, 32].

Lazarus (1991) and Archvadze (2021) showed that emotions can trigger different types of attitudes and behaviors. Fear, for example, would stimulate problem-solving or avoidance behaviors to prevent the feared incident or situation from occurring [33, 34].

Emotions are commonly used in communication and health education. Fear, anger and empathy are important levers for changing human behavior to an illness. Resilience and fatalism depend on the intensity of these emotions.

The fear of cancer controls the attributes of its perception, i.e. the label given to the disease, its causes and its consequences. It modulates the perception of controllability against carcinogenic factors and the preventive actions to be taken.

Being aware of emotions and their effects on our choices, decisions and preventive measures is one of the missions (=objectives) of health education.

The CSM of self-regulation delineates cognitive and emotional processes influencing motivations to engage in adaptive behaviors. Originally developed to account for reactions to health-related threats, this model has also utility for interventions to change behaviors in other domains involving threats [35].

In this study, the CSM allowed us to design our questionnaire items and analyze the perceptions of Tunisian youths about leukemia (Tables 1 and 2).

**Table 2:**
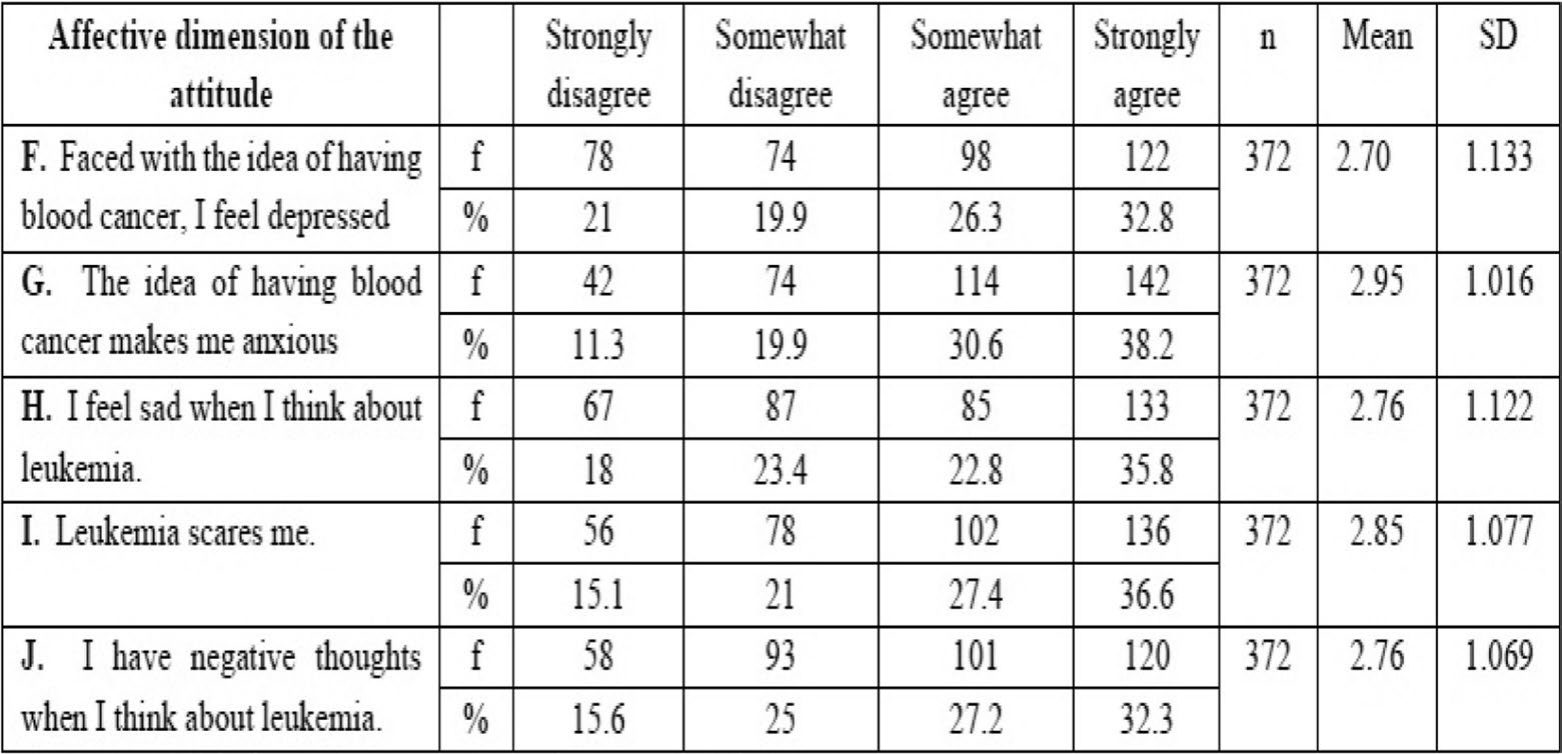
Participants’ level of agreement with statements in the affective dimension of attitudes toward leukemia

*SD* standard deviation, *f* frequency, *%* percentage student responses

### Environmental health literacy and empowerment perspectives on leukemia

Hill et al. (2010) studied the effects of cancer education on adults’ knowledge and attitude levels. They found that the participants’ levels of knowledge increased after receiving cancer education. In addition, the education program changed participants’ fatalistic attitudes towards cancer, with some beginning to behave more proactively towards cancer after receiving cancer education [8]. Thus, research indicates that education would have a significant effect on young people’s knowledge and attitudes towards cancer. Therefore, we believe that the development of health promotion interventions may be effective to prevent leukemia.

Furthermore, environmental health literacy is an evolving discipline that “combines key principles and procedural elements from various fields, such as risk communication, health literacy, environmental health sciences, communication research, and safety culture.” [36].

In light of the studies carried out by Finn and O’Fallon (2015), it would appear that the basic level of environmental health literacy will help people develop a sense of autonomy and self-efficacy by taking into account the environmental factors associated with leukemia and fulfill their roles as conscious and responsible actors in their own and others’ health.

In Tunisia, no leukemia curriculum has been included neither in physical courses nor in scientific and technological courses in primary and secondary schools. Aside from some information about the effects of tobacco and pollution, primary and secondary education curricula do not offer any scientific learning about cancer in life sciences, physics and chemistry.

We believe that the data collected from the study of the leukemia representations of Tunisian youths, their levels of environmental health literacy, and their perceptions of risk factors associated with leukemia could form the basis for designing a global strategy to promote leukemia prevention in Tunisia. The improvement of school curricula as well as educational and pedagogical interventions, and the establishment of a skill benchmark that meets our study’s objectives would not only increase the individuals’ power act confronted with the emergence of potentially leukemogenic environmental factors, but also help Tunisian youths adopt proactive behaviors that prevent leukemia and other cancers.

Three major research questions were identified as follows:

1. What are the secondary school students’ levels of knowledge about leukemogenic factors?
2. What are the students’ affective and behavioral attitudes towards leukemia?
3. What are the aspects of cancer in which secondary school students are most interested?

## Methods

### Research design and participants

This is a quantitative study based on a cross-sectional survey for data collection. We used random sampling to collect the required data from four public secondary schools, from the north, center and south of Tunisia. Data were gathered via a paper-and-pencil questionnaire inspired by the studies conducted by Heuckmann and Asshoff (2014) [37] and Yildirim-Usta (2020) [38] about cancer.

We have adapted the questionnaire to the specific case of leukemia.

A scientific team at the Pasteur Institute in Tunis, Tunisia checked the scientific relevance of questions and references. We tested the questionnaire (The language of teaching biology in Tunisia) based on a sample of students (15–19 years).

We noticed some linguistic difficulties. So, we added an Arabic translation. After a second test, we distributed the questionnaire.

Our sample includes students belonging to scientific and literary sections, where the percentage of female students is twice as high as that of males [39].

The participants belonged to low- and middle-income social categories, 100% were attending public schools (Table 3).

**Table 3:** The structure of the sample obtained from secondary school students in terms of Gender, School level and Region of residence for an age group (15–20 years) (n = 372)

Total		Numbers	% Percentages
		372	100
Gender	Male	119	32
	Female	253	68
School level	2nd	110	29,6
	3rd	141	37,9
	4th	121	32,5
Region	Ben Arous	114	30,6
	Seliana	37	9,9
	Kairouan	144	38,7
	Tataouine	77	20,7

Biology teachers were contacted to verify their availability and readiness to distribute the questionnaire in classes under the supervision of the principal investigator. 372 secondary school students participated in the study (68% female students; 32% male students; age range 16–20 years).

Before completing the questionnaire, the participants were informed of the anonymity and voluntary principles of this investigation. They were assured that they could stop the questionnaire at any time without any repercussions.

The study was approved by the ethics committee of the Pasteur Institute in Tunis (reference: 2020/23/I/LR16IPT), by the directors of the four secondary schools and the participants’ parents.

### Instruments

The data collection questionnaire consisted of four different parts:

The first part comprised questions related to demographic information, regarding gender, age and region of residence. The second part included a 12-statement dichotomous (yes/no) scale that was used to assess participants’ level of knowledge about leukemogenic factors. Participants were asked if they perceived a relationship between cancer and the statements in the questionnaire. They responded either “yes” or “no” to each statement [38].

The third part involved 12 items that assessed the participants’ attitudes towards cancer on a 4-point Likert-type scale (1 = strongly disagree, 2 = disagree, 3 = agree, and 4 = strongly agree). This section was divided into two components: affective and behavioral. Affective components were made up of five items (α =. 753) that evaluated negative emotional reactions towards leukemia. Behavioral components, however, contained seven items (α =. 756) that assessed proactive behaviors towards leukemia. These behavioral components were divided into three sub-sections, namely communication (three items; α =. 735), lifestyle choices (two items; α =. 949), and social engagement (two items; α =. 797).

The fourth part of the questionnaire included 9 items that tested students’ interest in leukemia through multiple types of cancer, risk factors, biological processes and the development of cancer treatment methods. Participants responded to the questionnaire using a 4-point Likert-type scale (1 = not interesting, 2 = somewhat interesting, 3 = interesting, and 4 = very interesting). Reliability was analyzed using Cronbach’s alpha, which was α =. 836.

### Data analysis

We used the statistical package for social sciences (SPSS, v.25.0) to analyze the collected data. First, we computed normality analyses for items located in the second (i.e., Level of knowledge about cancer risk factors), third (i.e., attitudes towards leukemia), and fourth (i.e., interest in leukemia) parts of the questionnaire. Based on the results of our normality analyses, we then used parametric tests to evaluate each research question.

All items were analyzed using descriptive statistics (i.e., mean values, standard deviations, frequencies, and percentages). In this study, the mean values of the items in the third and fourth parts of the questionnaire provided information regarding high school students’ opinions about leukemia. That is, mean values higher than 2.5 indicated that more than 50% of the participants tended to agree with or were interested in the statement. Mean values smaller than 2.5 indicated that less than 50% of participants tended to disagree with or were not interested in the statement, given that we utilized a 4-point rating scale (1 = strongly disagree to 4 = strongly agree; 1 = not interesting to 4 = very interesting) [38].

In SPSS, mean values were calculated based on the following formula to assess students’ attitudes and interests in leukemia:

([number of students who chose the response strongly disagree/not interesting] × [weighting of the response strongly disagree/not interesting] + [number of students who chose the response strongly disagree/somewhat interesting] × [weighting of the response strongly disagree/somewhat interesting] + [number of students who chose the answer agree / interesting] × [weighting of the response agree / interesting] + [number of students who chose the response strongly agree / very interesting] × [weighting of the response strongly agree / very interesting]) / (the total number of students who answered the questions) [38].

## Results

### Students’ level of knowledge about leukemogenic factors

**Fig. 1:**
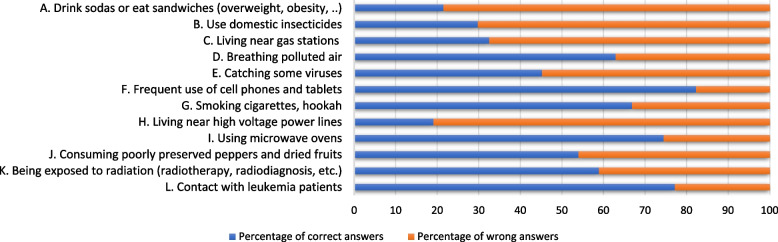
Percentages of students who identified the statement as leukemogenic

Secondary school students did not identify all leukemogenic factors (Fig. 1).

More specifically, most participants answered items D (62.9%), G (66.9%) and K (58.9%) correctly. However, many participants responded poorly to items A (78.5%), B (70.2%), C (67.5%) and H (80.9%) (obesity, household insecticides and proximity to service stations or high voltage power lines are not leukemogenic factors).

In addition, most participants were successful at identifying non-leukemogenic factors, as most responded correctly to items F (82.3%), I (74.5%), J (54%) and L (77, 2.5%).

### Students’ emotional attitudes towards leukemia

As shown in Table 2, the majority of participants agreed with the affective dimension statements (M = 2.80). The mean values of items F, G, H, I and J were greater than 2.5 (M = 2.70 / 59.1%; 2.95 / 68.8%; 2.76 / 58.6%; 2.85 / 64%; 2.24 / 59.5%, respectively). Many participants agreed with the statements.

### Students’ behavioral attitudes towards leukemia

As shown in Table 4, more than 50% of students tended to agree with items in the communication subsection (M, N, and O) of the behavioral dimension (M = 3.04).

**Table 4:**
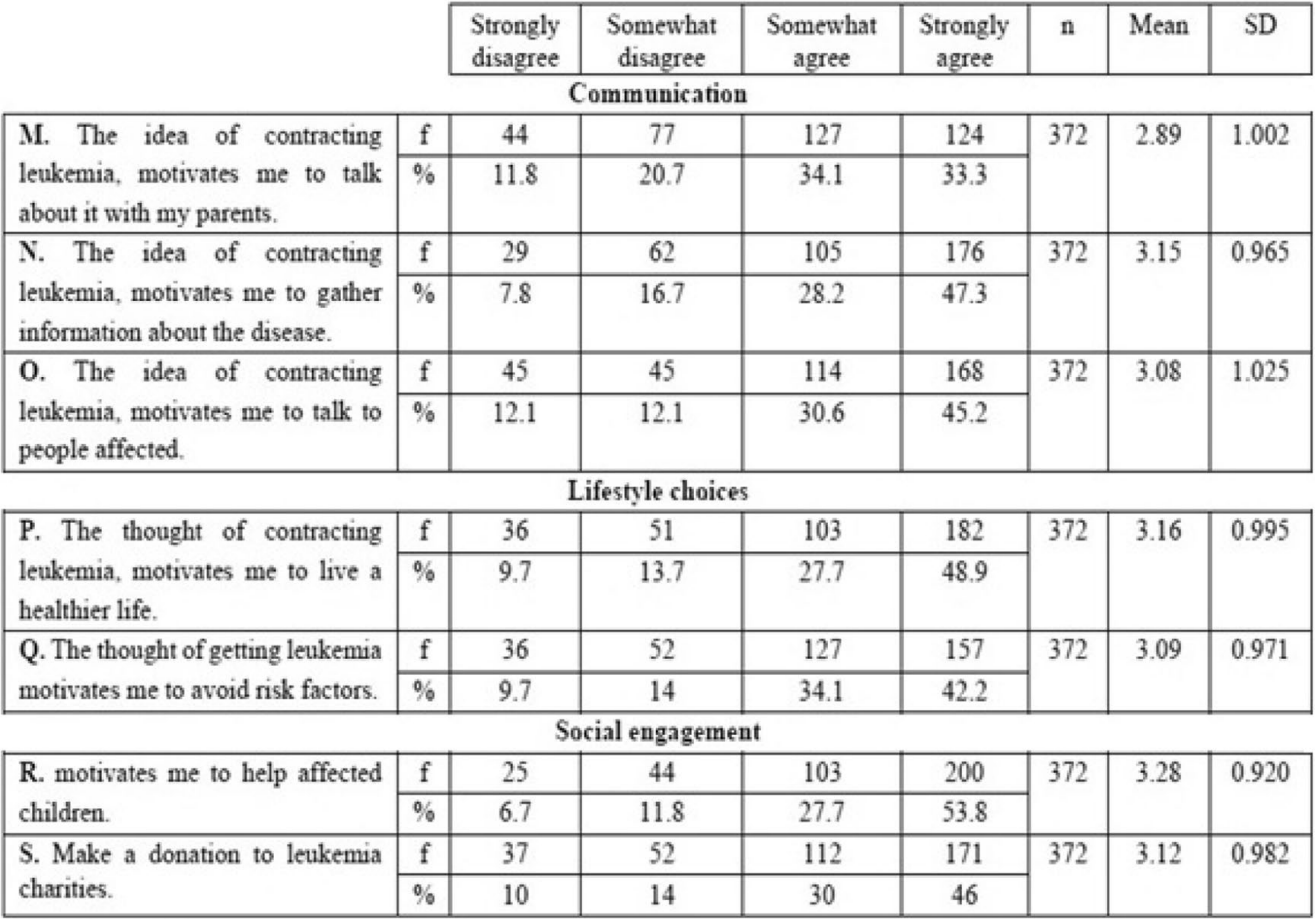
Participants’ level of agreement with statements in the behavioral dimension of attitudes toward leukemia

Similarly, most participants agreed with the statements in the lifestyle choices subsection (M = 3.12), with mean values for items P and Q being 3.16 and 3.09, respectively. Many participants agreed with these items (76.6 and 76.3%; *n* = 372).

For the social engagement subsection (M = 3.20), the mean values of items R and S were 3.28 and 3.12, respectively. Many participants agreed with these items (81.5 and 76%; n = 372).

### Students’ interest in cancer and leukemia

As shown in Table 5, students’ interest in cancer varied depending on the type of cancer, with the highest mean values occurred for items G (M = 3.20) and F (M = 3.15). Many participants were interested/very interested in both items (79.3 and 75.5%, *n* = 372).

**Table 5:**
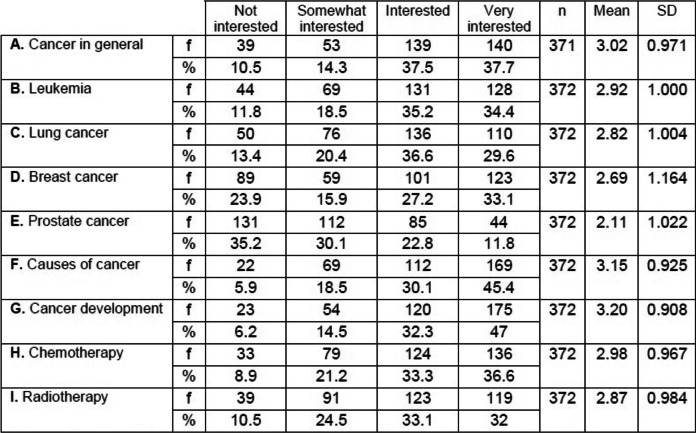
Students’ interest in learning about cancer

*SD* standard deviation, *f* frequency, *%* percentage of secondary school student’s responses

Participants were clearly interested/very interested in leukemia i.e., item B (M = 2.92) with (69.6%, n = 372).

The lowest mean value occurred for item E (M = 2.11). Many participants were not / rather interested in prostate cancer (65.3%, n = 372 with 68% female, 32% male). Breast and prostate cancers are subjects of heterogeneous interest, depending on the individual’s gender.

## Discussion

Primary cancer prevention involves reducing exposures to risk factors or modifying the underlying conditions that lead to the disease.

Recent history is rich with examples of harmful, unnecessary, and preventable exposures where scientific uncertainty and lack of consensus have resulted in health and environmental problems including certain chemicals such as DDT, PCBs, and lead used decades after the first warning signs [40]. Currently, the European Community recognizes the precautionary principle, which justifies public policy actions in situations of scientific uncertainty in order to reduce threats to health.

In contrast, the regulatory framework in other countries generally requires scientific and biomedical consensus on the evidence of harm before any policy action is taken.

In Tunisia, the lack of school curricula and public health campaigns specifically focused on primary prevention of childhood leukemia may, in part, result from this tendency to avoid taking precautionary measures without causal evidence.

Yet, each of these potential environmental leukemogenic factors is also associated with other health problems in childhood and adulthood. In fact, these exposures that are implicated in the risk for childhood leukemia have substantial documentation of non-cancer health impacts, including neurobehavioral deficits and respiratory disorders [7].

Thus, actions taken to prevent childhood leukemia would also reduce the incidence of other diseases, and vice versa. Parental smoking, for instance, is already the subject of public health recommendations. In this context, the inclusion of childhood leukemia in the existing public health campaigns may improve the effectiveness of these promotion interventions.

In Tunisia, with the exception of tobacco control and some aspects of pollution, educational efforts for large-scale promotion of environmental health activities remain limited despite the fact that the co-benefits of primary prevention extend beyond leukemia to other comorbidities associated with the same exposures.

### Students’ level of knowledge about leukemogenic factors

Our study showed that the majority of participants failed to identify leukemogenic risk factors (Fig. 1), with more than 74% of participants stating that there was no relationship between the use of domestic insecticides, obesity, proximity to heavy-traffic roads, benzene exposure or high-voltage power lines and blood cancer.

Our results confirm the findings of Heuckmann and Asshoff (2014) [37] and Yildirim-Usta (2020) [38] in which learning about cancers in schools led to the identification of carcinogenic factors by Turkish and German students.

In recent years, obesity has been recognized as a factor in the development of various cancers. Indeed, endometrial, postmenopausal breast, and colorectal cancers accounted for 65% of these cancers [41].

Recent reviews and meta-analyses have recently highlighted the role of obesity in increasing the prevalence of various hematologic cancers [42–45].

78% of the participants did not identify the link between obesity and leukemia. This result confirms Karayurt et al. (2008) [46] and Yildirim-Usta (2020) [38] studies, where students reported that there was no relationship between cancer and overweight. This knowledge gap is explained by the absence of information concerning the relationship between cancer and obesity in the Tunisian Life Sciences curriculum. During their school years, students were taught about the importance of a balanced diet on several occasions, in the ninth year of primary school (Unit 2: Nutritional functions) [47], the second year of secondary school (Unit 1: Food hygiene) and the third year of secondary school (Unit 1: Nutrition and health). However, the carcinogenic consequences of obesity were not mentioned [48].

In addition, the same respondents identified smoking and polluted air as leukemogens. Frequent and prolonged exposure to informative and inciting content on the dangers of tobacco in the media and through education had certainly contributed to a more sensitive perception of this factor, compared to other less known factors by the general public.

This result is consistent with the findings of Oakley et al., (1995) [49], who asked participants to name carcinogenic factors of which they were already aware. Most respondents noted that smoking is a carcinogenic factor. Similarly, Knighting et al. (2011) [50] studied children’s understanding of cancer by asking participants to draw and write anything about cancer. They found that smoking was the most described carcinogenic factor.

During their school years, Tunisian youths got massive amounts of information about the dangers of pollution which explain their sensitivity to this leukemogenic factor and its qualification. However, the same students were unable to assess the risks of exposure to chemical pollutants whether by inhaling domestic insecticides or by living near a gas station. They were also unable to assess the risks of exposure to low-frequency electromagnetic radiation by living near high-voltage power lines.

These difficulties are explained by non-updated school curricula including new scientific knowledge. The last Tunisian reform in biology education dates back to 2009. Its adequacy with the scientific progress constitutes an absolute urgency in particular in order to achieve awareness of the environmental risk factors associated with blood cancers.

Preventive actions suggested by research on leukemia evoke not only the need for smoking cessation, healthy eating during pregnancy and folate supplementation, but also the avoidance of exposure to volatile organic compounds, pesticides and paintings [51].

The adherence, acceptability and effectiveness of these precautionary and preventive measures depend on improving environmental health literacy. Program designers should understand the importance of learning about environmental risk factors associated with cancers.

There are several exploration paths, such as providing information on leukemia risks due to the excessive use of insecticides by farmers in the first year of the secondary school (Unit 1: Improving Crop Production), programming learning sequences related to pollution and leukemogenic environmental factors in the second year of the scientific pathway (Unit 2: Rational Management of Ecosystems), focusing on cancer education courses as well as mutagenic and carcinogenic factors, like radiation and certain chemicals (e.g. benzopyrene and nitrosamines) in the third year of the scientific and literary pathways (Unit 2: Genetic information and its expression), and investigating awareness of the role of folate in the prevention of childhood leukemia during pregnancy in the fourth year of scientific and literary pathways (Unit 1: Human Reproduction and Health).

A deep understanding of the fundamental mechanisms and causes of cancer depends on the way it is taught in biology and other related disciplines, like physical sciences in which the awareness of radiation and cancer risks as well as electromagnetic fields can be highlighted in the third and fourth years of the scientific pathway.

### Students’ attitudes towards leukemia in the affective and behavioral dimension

#### Affective dimension

Most students agreed with all affective dimension items regarding attitudes toward leukemia (i.e., most respondents exhibited negative emotions when they thought about leukemia (Table 2).

The perception of controllability, prevention and curability of this disease among most participants was low. Fatalism is associated with the perceived identity of blood cancer as a critical disease with severe and often fatal consequences.

There is no doubt that the lack of information (=knowledge) about cancer treatment in education programs including biology and other related disciplines (e.g. physics and chemistry) explains these results. However, the results of Heuckmann and Asshoff (2014) [37] and Yildirim-Usta (2020) [38] reveal that, despite the introduction of information on this topic, most German and Turkish students experienced negative emotions due to the sense of “uncontrollability” of cancer and its associated factors.

In light of these results, the design of a national didactic curriculum to support leukemia prevention should reconcile informative purposes and the development of psychosocial skills essential to a reasoned perception of cancer risks associated with environmental factors. In view of the emotional charges and persistent beliefs about cancer, the development of key competencies is crucial for students. Furthermore, the adoption of proactive behaviors in the face of leukemogenic factors depends on the teaching practices envisaged in the classroom. Investigative and project-based pedagogies constitute an interesting approach for a better understanding of the disease, its causes and possible means to reduce exposure risks.

Other more contextual factors may explain the dominance of negative emotions. Fate is a major belief that not only affects individuals’ cancer screening behaviors, but also poses a problem for its treatment [52].

Tunisia is an Arab-Muslim country in which faith in destiny is central. People’s behaviors are largely influenced by a dominant set of cultural and religious beliefs. Thus, statements such as “we cannot prevent cancer if it is in our destiny, or we have no real control over our lives because everything was already decided” are convictions often held in Muslim countries.

Preventive and curative approaches often come up against the erroneous and unfounded social acceptance of the notions of destiny and fatality.

The effectiveness of any preventive or curative interventions in these societies depends on a better understanding of these notions. Thus, the inclusion of preventive and therapeutic resilience by developing cross-cutting competencies to address cancer in religious education courses for primary and secondary schools would allow for a better understanding of the concepts (“destiny” and ‘fatality”), which would facilitate individuals’ adherence to preventive and therapeutic interventions.

#### Behavioral dimension

Regarding the behavioral dimensions of leukemia (Table 4), the majority of participants agreed with items in the communication subsection. This result is in sharp contrast with Yildirim-Usta’s (2020) study [38], where more than half of the respondents were not motivated to talk to their parents about cancer or obtain information about the disease.

Furthermore, most Tunisian students agreed with statements from the lifestyle choices and social engagement subsections. This outcome is consistent with the findings of Heuckmann and Asshoff (2014) [37]. In particular, the idea of contracting leukemia motivated most youth to lead a healthy life, change their lifestyle, and help children with blood cancer.

### Students’ interest in learning about leukemia

Tunisian students expressed a high level of interest in learning about cancer (Table 5). This finding is congruent with Heuckmann and Asshoff’s (2014) findings [37].

Topics which participants found interesting in our study and the studies conducted by Heuckmann and Asshoff (2014) [37] and Yildirim-Usta (2020) [38] included leukemia, other cancers and their development. Moreover, there is growing interest in learning more about risk factors and treatment methods. Differences are probably explained by the presence of information on these topics in German and Turkish biology and health courses.

Heuckmann and Asshoff (2014) [37] suggested that students’ interest in learning more about cancer will stimulate communication about this disease in classrooms. Thus, examples of cancers that have generated the greatest interest among students can be included in Tunisian life science curricula. Based on our findings, cancer-specific topics can be included in biology courses. Anchoring information on the causes, symptoms, prevention, diagnosis and treatment of cancer in life science curricula for both primary and secondary schools is not only possible but necessary in order to satisfy knowledge needs expressed by Tunisian youths.

Several paths for anchoring information on cancer remain to be explored. For instance, raising awareness on the importance of imaging techniques for diagnosing cancers, such as brain tumors, could be highlighted in the “Nervous system hygiene” theme in the ninth year of primary school and the fourth year of secondary school. Current treatment methods could be addressed in the “Conformal Reproduction” topic taught in the second year of secondary education. New treatment methods could be included in the “Biotechnology and Genetic Engineering” topic taught in the third year of secondary school.

### Cancer education: a systemic approach to individual and social change

For primary prevention and reduction of the prevalence of leukemia in Tunisia and other countries, it is relevant to target modifiable or avoidable risk factors. For example, paternal smoking, exposure to pesticides and nitrogen dioxide, are identified in the literature [53].

The anticipation of environmental carcinogenic factors by individuals requires cancer education at an early age. This education must be not only cross-disciplinary and multidisciplinary, but also part of the ecological approach to health and sustainable development.

Knowledge acquisition will reduce uncertainty associated with cancer.

The development of various skills, involving psychosocial skills, critical thinking, autonomy and communication, will provide youths with suitable means to anticipate and prevent certain risks. Constructs, such as awareness, changes in attitudes and the adoption of new behaviors require time and upstream work in schools.

Cancer education should inspire initiatives for change through individuals’ actions to avoid smoking, haphazard use of pesticides and pollution. But it is also a citizenship education that has the potential to put health and environmental concerns at the center of political and media debates, especially in developing countries. If the social demand for reforms is strong enough, systemic change will then be possible.

### Limitations

For feasibility reasons, sampling was restricted to four secondary public schools in the North, Center and South of Tunisia. A large-scale investigation could confirm our findings.

Furthermore, our results based on declarative data collected by teachers and their students, were therefore, subject to social desirability bias.

## Conclusion

Environmental health literacy is the result of the interaction between a person’s abilities: to recognize the need for environmental health information, to find that information, to understand it, and to use it, as well as, to make informed decisions about their environment and health. This study reveals lack of knowledge about leukemia and its environmental factors.

Low scores and students’ strong interests in information should encourage educational actors to integrate cancer education into Tunisian Life Science curricula. Indeed, the adherence, acceptability and effectiveness of these precautionary and preventive measures depend on improving environmental health knowledge.

From the perspective of empowerment through health education, the emergence of a reasoned individual perception in schools in the face of unpredictable environmental factors and biomedical uncertainties depends on the development of both psychosocial skills and critical thinking skills capable of managing complex situations and making well-thought-out health choices.

## Data Availability

The datasets used and/or analyzed during the current study are available from the corresponding author on reasonable request.
